# Sulfonylurea Is Associated With Higher Risks of Ventricular Arrhythmia or Sudden Cardiac Death Compared With Metformin: A Population‐Based Cohort Study

**DOI:** 10.1161/JAHA.122.026289

**Published:** 2022-09-14

**Authors:** Teddy Tai Loy Lee, Jeremy Man Ho Hui, Yan Hiu Athena Lee, Danish Iltaf Satti, Yuki Ka Ling Shum, Pias Tang Hoi Kiu, Abraham Ka Chung Wai, Tong Liu, Wing Tak Wong, Jeffrey Shi Kai Chan, Bernard Man Yung Cheung, Ian Chi Kei Wong, Shuk Han Cheng, Gary Tse

**Affiliations:** ^1^ Department of Emergency Medicine School of Clinical Medicine, The University of Hong Kong Hong Kong China; ^2^ Diabetes Research Unit, Cardiovascular Analytics Group China‐UK Collaboration Hong Kong China; ^3^ Tianjin Institute of Cardiology, The Second Hospital of Tianjin Medical University Tianjin China; ^4^ School of Life Sciences, State Key Laboratory of Agrobiotechnology (CUHK), The Chinese University of Hong Kong Hong Kong China; ^5^ Division of Clinical Pharmacology School of Clinical Medicine, The University of Hong Kong Hong Kong China; ^6^ Department of Pharmacology and Pharmacy University of Hong Kong Hong Kong China; ^7^ UCL School of Pharmacy Medicines Optimisation Research and Education (CMORE) London United Kingdom; ^8^ Department of Infectious Diseases and Public Health City University of Hong Kong Hong Kong China; ^9^ Kent and Medway Medical School Canterbury United Kingdom

**Keywords:** metformin, sudden cardiac death, sulfonylurea, type 2 diabetes, ventricular arrhythmia, Ventricular Fibrillation, Arrhythmias, Sudden Cardiac Death

## Abstract

**Background:**

Commonly prescribed diabetic medications such as metformin and sulfonylurea may be associated with different arrhythmogenic risks. This study compared the risk of ventricular arrhythmia or sudden cardiac death between metformin and sulfonylurea users in patients with type 2 diabetes.

**Methods and Results:**

Patients aged ≥40 years who were diagnosed with type 2 diabetes or prescribed antidiabetic agents in Hong Kong between January 1, 2009, and December 31, 2009, were included and followed up until December 31, 2019. Patients prescribed with both metformin and sulfonylurea or had prior myocardial infarction were excluded. The study outcome was a composite of ventricular arrhythmia or sudden cardiac death. Metformin users and sulfonylurea users were matched at a 1:1 ratio by propensity score matching. The matched cohort consisted of 16 596 metformin users (47.70% men; age, 68±11 years; mean follow‐up, 4.92±2.55 years) and 16 596 sulfonylurea users (49.80% men; age, 70±11 years; mean follow‐up, 4.93±2.55 years). Sulfonylurea was associated with higher risk of ventricular arrhythmia or sudden cardiac death than metformin hazard ratio (HR, 1.90 [95% CI, 1.73–2.08]). Such difference was consistently observed in subgroup analyses stratifying for insulin usage or known coronary heart disease.

**Conclusions:**

Sulfonylurea use is associated with higher risk of ventricular arrhythmia or sudden cardiac death than metformin in patients with type 2 diabetes.

**Nonstandard Abbreviations and Acronyms**
SCD: sudden cardiac death
VA: ventricular arrhythmia

**Clinical Perspective**

**What Is New?**

- Sulfonylurea use is associated with a higher risk of ventricular arrhythmias and sudden cardiac death compared with metformin use.
- Our results show higher incidence of ventricular arrhythmias and sudden cardiac death in tolbutamide users compared with other sulfonylureas.

**What Are the Clinical Implications?**

- The findings of this study highlight the importance of moving away from using sulfonylureas in type 2 diabetes control.

Type 2 diabetes is a highly prevalent condition worldwide; 1 in 11 adults globally have type 2 diabetes. Every year, it causes over 1 million deaths, making it the ninth leading cause of mortality.^1^ Cardiovascular complications are significantly associated with death among patients with type 2 diabetes,^2^ with ventricular arrhythmia and sudden cardiac death (VA/SCD) being the most common macrovascular complications.^3^ Siscovick and colleagues estimated the incidence rate of sudden cardiac arrest to be 3.15 per 1000 patients with diabetes without prior clinically recognized heart disease; and the incident rate was 13.80 per 1000 patients with diabetes with clinically recognized heart disease, 3.84 and 2.31 times higher when compared with patients without diabetes, respectively.^4^ The elevated risk can be attributed to a poor glycemic control and other risk factors such as dyslipidemia and nephropathy.^4^

Metformin and sulfonylurea were frequently prescribed because of their effective glycemic control and low cost even though their cardiovascular risks continue to be debated.^5^ The effect of antidiabetic medications on atrial fibrillation has been well documented,^6^, ^7^ yet not many studies investigated the effect of such medications on the risk of VA/SCD, let alone the comparison between metformin and sulfonylurea use. There is a need for the above investigation as sudden cardiac death, the most devastating manifestation of VA, is the leading cause of death among patients with type 2 diabetes with cardiovascular complications.^8^

Therefore, this study aimed to compare the risk of developing VA/SCD between metformin and sulfonylurea users.

## Methods

### Data Source

This study has been approved by the Joint Chinese University of Hong Kong–New Territories East Cluster Clinical Research Ethics Committee. This was a population‐based retrospective cohort study. Data were extracted from the Clinical Data Analysis and Reporting System, a territory‐wide electronic database storing patient health care records managed by the Hong Kong Hospital Authority, which manages 42 hospitals, 47 specialist outpatient clinics, and 73 general outpatient clinics, serving a population of 7 million. This database has previously been used for cohort studies by local teams in Hong Kong.^9^, ^10^, ^11^ The need for informed consent was waived because of the observational nature and the use of deidentified data in this study. The data that support the findings of this study are available from the corresponding author upon reasonable request.

### Study Cohort

Patients who fulfilled all of the following inclusion criteria were included: (1) aged ≥40 years; and (2) had documented diagnosis of type 2 diabetes under the *International Classification of Diseases, Ninth Revision* (*ICD‐9*) coding system, or were prescribed antidiabetic agents between January 1, 2009, and December 31, 2009. Patients who were prescribed both metformin and sulfonylurea or had past medical history of myocardial infarction, as identified using *ICD‐9* codes, were excluded from the study.

### Outcomes

The primary outcome of the study is a composite of ventricular arrhythmia and sudden cardiac death (VA/SCD), as identified using *ICD‐9* codes. A diagnosis of VA/SCD was made on the basis of clinical judgment by the treating physician and was then coded according to the *ICD‐9* system into the Clinical Data Analysis and Reporting System. VA/SCD episodes in which a myocardial infarction occurred within 1 week before or after the VA/SCD episode were considered acute myocardial infarction related and thus excluded. Patients were followed up until December 31, 2019.

### Propensity Score Matching

To facilitate comparability of the cohorts, propensity score matching was performed with the baseline demographics of sex, age, and duration since type 2 diabetes diagnosis; prior comorbidities including peripheral vascular disease, ischemic stroke, atrial fibrillation, heart failure, prior VA/SCD, intracranial hemorrhage, coronary heart disease, hypertension, and chronic obstructive pulmonary disease; baseline medication use including angiotensin‐converting enzyme inhibitors, beta blockers, calcium channel blockers, diuretics, and use of diabetic medications including insulin, thiazolidinediones, dipeptidyl‐peptidase 4 inhibitors, and glucagon‐like peptide agonists; and laboratory tests of hemoglobin A_1c_. The *ICD‐9* codes used for classification of the corresponding medical conditions are available in Table S1.

Imputation was not performed; patients with missing data were excluded during the generation of the matched cohort. Patients with use of either metformin or sulfonylurea were matched on a 1:1 ratio on the propensity score, which was derived from logistic regression using the nearest‐neighbor matching algorithm. To assess the balance of covariates, the standardized mean difference, which is the difference in means or proportions over the pooled SD, was used for categorical covariates, while the variance ratio, which is the ratio of variance between the treatment and control groups, was used for both categorical and continuous covariates. A caliper width of 0.2 was chosen as it is considered as the optimal caliper width for estimating mean differences.^12^ Covariates were considered balanced in both groups when the standardized mean difference was <0.1, and when the variance ratio was between 0.5 and 2.0.^13^

### Statistical Analysis

Continuous variables were presented as the mean (SD), and categorical variables were presented as frequency (%). Hazard ratios (HRs), along with 95% CIs and *P* values were reported accordingly with the univariate Cox proportional hazards regression model. The proportional hazards assumption of the model was assessed by performing the Schoenfeld proportionality test; the results indicated that the assumption was met. Kaplan‐Meier curves were plotted against the time‐to‐event for VA/SCD stratified by either metformin or sulfonylurea use. The log‐rank test was performed to investigate the statistical significance between the metformin and sulfonylurea groups. Incidence rates for VA/SCD were presented as incidence per 1000 person‐years along with 95% CIs. Subgroup analyses were conducted to investigate the risk of VA/SCD by users versus nonusers of insulin and patients with versus without prior coronary heart disease, and by different types of sulfonylurea. Sensitivity analysis was performed by excluding patients with prior cardiomyopathy or valvular disease and excluding patients with prior heart failure. Computed E‐values for HRs were computed to quantify the effects of any unmeasured confounding on our study. The E‐value is defined as the minimum strength of association that an unmeasured confounder would be required to have with both the outcome and treatment to fully explain away a specific association between the treatment and outcome, conditional on the measured covariates.^14^ In this study, a large E‐value implies that an unmeasured confounder must be very strong to explain away the effect of sulfonylurea over metformin use in the risk of developing VA/SCD.

All probability tests were 2‐tailed and considered significant at *P*<0.05. No imputation was performed for missing data. Statistical analyses were conducted using RStudio 1.4.1717 (RStudio, Boston, Massachusetts).

## Results

**Figure 1:**
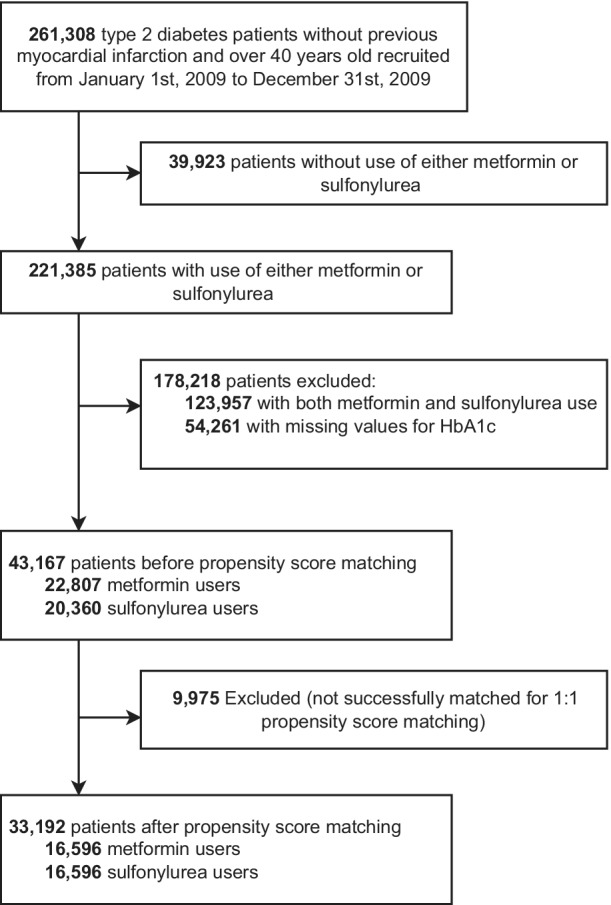
Study flowchart.

The steps for the selection of patients for this study cohort are shown in Figure 1. In total, 261 308 patients fulfilled the inclusion criteria. After excluding patients with missing data, the study cohort consisted of 43 167 patients (47.3% men; mean baseline age, 68±12 years), where 22 807 (52.83%) patients received metformin and 20 360 (47.17%) patients received sulfonylurea. After 1:1 propensity score matching, the final study cohort consisted of 16 596 metformin users and 16 596 matched sulfonylurea users. The baseline and clinical characteristics of the study population before and after propensity score matching are shown in Table 1. A Love plot summarizing covariate balances before and after propensity score matching is provided in Figure S1. Standardized mean difference values were <0.1 and variance ratio values were within 0.5 to 2.0, indicating a good balance in baseline characteristics between the two cohorts; the only exception was age, which had a standardized mean difference of 0.13, but the variance ratio value was 1.09, which was very close to 1.0, indicative of good balance.^15^

**Table 1:** Baseline Characteristics Before and After 1:1 Propensity Score Matching

Characteristics	Before matching		After matching			
	Metformin (n=22 807)	Sulfonylurea (n=20 360)	Metformin (n=16 596)	Sulfonylurea (n=16 596)	SMD	VR
Demographics						
Male, n (%)	9797 (43.0)	10 619 (52.2)	7911 (47.7)	8272 (49.8)	0.02	…
Baseline age, y	65.17±11.61	71.31±11.39	68.14±10.89	69.60±11.35	0.13	1.09
Follow‐up duration since type 2 diabetes diagnosis, y	4.98±2.56	4.92±2.55	4.92±2.55	4.93±2.55	<0.01	1.00
Comorbidities						
Peripheral vascular disease, n (%)	61 (0.3)	29 (0.1)	29 (0.2)	27 (0.2)	<0.01	…
Ischemic stroke, n (%)	971 (4.3)	1419 (7.0)	859 (5.2)	986 (5.9)	<0.01	…
Atrial fibrillation, n (%)	775 (3.4)	1547 (7.6)	734 (4.4)	966 (5.8)	0.01	…
Heart failure, n (%)	814 (3.6)	2258 (11.1)	795 (4.8)	1259 (7.6)	0.03	…
Prior VA/SCD, n (%)	4 (0.0)	11 (0.1)	4 (0.0)	7 (0.0)	<0.01	…
Intracranial hemorrhage, n (%)	322 (1.4)	517 (2.5)	306 (1.8)	362 (2.2)	<0.01	…
Coronary heart disease, n (%)	2480 (10.9)	3618 (17.8)	2188 (13.2)	2529 (15.2)	0.02	…
Hypertension, n (%)	6947 (30.5)	8964 (44.0)	5904 (35.6)	6537 (39.4)	0.04	…
Chronic obstructive pulmonary disease, n (%)	60 (0.3)	135 (0.7)	58 (0.3)	90 (0.5)	<0.01	…
Medications						
ACE inhibitors, n (%)	12 795 (56.1)	11 141 (54.7)	8848 (53.3)	9025 (54.4)	0.01	…
Beta blockers, n (%)	8988 (39.4)	8372 (41.1)	6838 (41.2)	6837 (41.2)	<0.01	…
Calcium channel blockers, n (%)	9598 (42.1)	9997 (49.1)	7529 (45.4)	7810 (47.1)	0.02	…
Diuretics, n (%)	4382 (19.2)	6096 (29.9)	3761 (22.7)	4292 (25.9)	0.03	…
Insulin, n (%)	6616 (29.0)	2661 (13.1)	2429 (14.6)	2501 (15.1)	<0.01	…
Thiazolidinediones, n (%)	260 (1.1)	225 (1.3)	169 (1.0)	176 (1.1)	<0.01	…
DPP4 inhibitors, n (%)	45 (0.2)	11 (0.1)	12 (0.1)	10 (0.1)	<0.01	…
GLP‐1 agonists, n (%)	9 (0.0)	0 (0.0)	0 (0.0)	0 (0.0)	<0.01	…
Glicazide, n (%)	…	13 093 (64.3)	…	10 390 (62.6)	…	…
Glipizide, n (%)	…	669 (3.3)	…	516 (3.1)	…	…
Tolbutamide, n (%)	…	698 (3.4)	…	489 (2.9)	…	…
Glibenclamide, n (%)	…	1559 (7.7)	…	1306 (7.9)	…	…
Glimepiride, n (%)	…	275 (1.4)	…	227 (1.4)	…	…
Laboratory tests						
Hemoglobin A_1c_, %	7.45±1.44	7.44±1.45	7.44±1.44	7.45±1.45	<0.01	1.02

Continuous variables were expressed as mean±standard deviation. SMD <0.1/VR>0.5 and <2.0 indicated good balance in matching. ACE indicates angiotensin‐converting enzyme; DPP4, dipeptidyl‐peptidase 4; GLP‐1, glucagon‐like peptide 1; SMD, standardized mean difference; VA/SCD, ventricular arrhythmia or sudden cardiac death; and VR, variance ratio.

**Figure 2:**
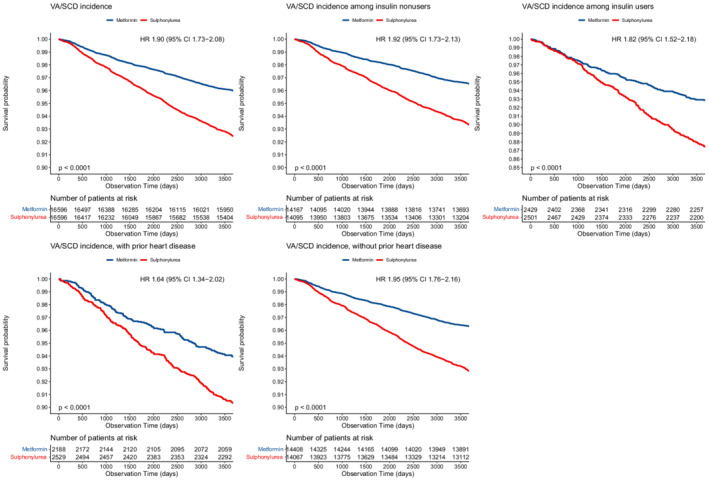
Kaplan‐Meier survival curves of VA/SCD stratified by metformin vs sulfonylurea from main and subgroup analysis. Blue=metformin, red=sulfonylurea. HR indicates hazard ratio; and VA/SCD, ventricular arrhythmia or sudden cardiac death.

The main and subgroup analysis of VA/SCD risk are presented in Table 2. The mean follow‐up duration was 4.92±2.55 years for the metformin cohort, and 4.93±2.55 years for the sulfonylurea cohort. During the study period, 711 metformin users and 1328 sulfonylurea users experienced episodes of VA/SCD. Cox proportional hazards model analyses over the entire follow‐up showed that sulfonylurea use was associated with an overall higher risk of VA/SCD than metformin use (HR, 1.90 [95% CI, 1.73–2.08]), as visualized by the Kaplan‐Meier curves in Figure 2. The corresponding E‐value was 3.21.

**Table 2:** Main, Subgroup, and Sensitivity Analysis of VA/SCD Risk in Sulfonylurea Over Metformin Users

Sulfonylurea vs metformin	HR (95% CI)	Metformin users			Sulfonylurea users			E‐value (HR)
		Cohort size	Number of events	Follow‐up person‐years/incidence per 1000 person‐years (95% CI)	Cohort size	Number of events	Follow‐up person‐years/incidence per 1000 person‐years (95% CI)	
Overall	1.90 (1.73–2.08)	16 596	711	81 682/8.70 (8.08–9.37)	16 596	1328	81 777/16.24 (15.38–17.14)	3.21
Without history of valvular heart disease/cardiomyopathy	1.97 (1.79–2.17)	15 426	619	75 837/8.16 (7.53–8.83)	14 992	1163	74 071/15.70 (14.81–16.63)	3.35
Without history of heart failure	1.94 (1.76–2.14)	15 801	620	77 605/7.99 (7.37–8.64)	15 337	1148	77 688/14.78 (13.93–15.66)	3.29
Insulin use								
Insulin users only	1.82 (1.52–2.18)	2429	181	12 021/15.06 (12.94–17.42)	2501	333	12 329/27.01 (24.19–30.07)	3.04
Without insulin use	1.92 (1.73–2.13)	14 167	530	69 660/7.61 (6.97–8.28)	14 095	995	69 448/14.33 (13.45–15.25)	3.25
Coronary heart disease								
Without coronary heart disease	1.95 (1.76–2.16)	14 408	571	70 602/8.09 (7.44–8.80)	14 067	1069	69 078/15.47 (14.56–16.43)	3.31
With coronary heart disease only	1.64 (1.34–2.02)	2188	140	11 080/12.64 (10.63–14.91)	2529	259	12 699/20.40 (17.99–23.04)	2.67

A large E‐value implies that any unmeasured confounder must be strong to explain away the effect of sulfonylurea over metformin use in the risk of developing VA/SCD. HR indicates hazard ratio; and VA/SCD, ventricular arrhythmia or sudden cardiac death.

Subgroup analyses were performed by concurrent use of insulin and the presence of coronary heart disease; sensitivity analysis was performed by excluding patients with either valvular heart disease or cardiomyopathy and by excluding patients with heart failure (Table 2). Baseline characteristics of the subgroups are summarized in Tables S2 through S4. Sulfonylurea was consistently associated with a higher risk of VA/SCD in patients without a history of valvular heart disease or cardiomyopathy (HR, 1.97 [95% CI, 1.79–2.17]), without a history of heart failure (HR, 1.94 [95% CI, 1.76–2.14]), and in both insulin users (HR, 1.82 [95% CI, 1.52–2.18]) and nonusers (HR, 1.92 [95% CI, 1.73–2.13]). Similarly, sulfonylurea use was associated with higher risks of VA/SCD across those with (HR, 1.64 [95% CI, 1.34–2.02]) and without (HR, 1.95 [95% CI, 1.76–2.16]) coronary heart disease.

The risk of VA/SCD between individual types of sulfonylurea is shown in Table 3. Glicazide (HR, 1.74 [95% CI, 1.57–1.93]), glipizide (HR, 2.15 [95% CI, 1.54–3.01]) and glimepiride (HR, 2.79 [95% CI, 1.82–4.29]) were significantly associated with a higher risk of VA/SCD, with tolbutamide being associated with the highest risk (HR, 4.70 [95% CI, 3.58–6.17]). The corresponding E‐values were 2.87, 3.72, 5.02, and 8.87, respectively. Baseline characteristics of individual sulfonylurea users are shown in Table S5.

**Table 3:** Subgroup Analysis of VA/SCD Risk by Individual Sulfonylurea

Sulfonylurea versus metformin	HR (95% CI)	Cohort size	Number of events	Follow‐up person‐years/incidence per 1000 person‐years	E‐value (HR)
Glicazide	1.74 (1.57–1.93)	10 116	744	49 983/14.89 (13.83–15.99)	2.87
Glipizide	2.15 (1.54–3.01)	399	36	1998/18.02 (12.62–24.94)	3.72
Tolbutamide	4.70 (3.58–6.17)	302	56	1503/37.26 (28.14–48.38)	8.87
Glibenclamide	1.16 (0.89–1.51)	1216	60	6157/9.75 (7.44–12.54)	1.00*
Glimepiride	2.79 (1.82–4.29)	180	22	829/26.54 (16.63–40.18)	5.02

A large E‐value implies that any unmeasured confounder must be strong to explain away the effect of sulfonylurea over metformin use in the risk of developing VA/SCD. HR indicates hazard ratio; and VA/SCD, ventricular arrhythmia or sudden cardiac death.

* The CI of the hazard ratio crossed 1; thus, its E‐value is 1.

## Discussion

The main finding of this study is that sulfonylurea users have a higher risk of ventricular arrhythmia and sudden cardiac death than metformin users. These results were consistent irrespective of severity of diabetes or history of coronary heart disease.

### Mechanism Underlying the Observations

It is hypothesized that sulfonylurea may inhibit the delayed rectifier potassium channel, leading to prolonged QT interval.^16^ A trial conducted in 30 patients with type 2 diabetes found that patients randomized to glyburide were associated with an increase in corrected QT interval compared with patients randomized to metformin.^17^ Sulfonylurea carries a high risk of hypoglycemia compared with other antidiabetic medications,^18^ in turn prolonging action potentials in myocardial tissue by blocking potassium channels at the cellular level.^19^ Multiple studies found that severe hypoglycemia, a major concern of sulfonylurea, increased the risk of VA/SCD.^20^, ^21^ However, sulfonylurea may also have antiarrhythmic effects by inhibiting reentrant arrhythmias by a mechanism known as ischemic preconditioning, and thus reducing the risk of developing cardiac arrest,^16^ but it has been suggested that the effects of ischemic preconditioning are abolished in type 2 diabetes.^22^ Metformin has pleotropic effects with many cardiovascular benefits as shown in basic science studies^23^, ^24^; it was found to be associated with a decreased corrected QT interval in animal models,^25^ but no decrease in ventricular arrhythmic outcomes was reflected in clinical trials.^26^

### Comparison With Previous Observational Studies

Two recent observational studies have investigated the association between metformin and sulfonylurea use and risk of VA/SCD.^27^, ^28^ Ostropolets and colleagues^27^ found that patients with diabetes on metformin monotherapy had a reduced risk of VA compared with sulfonylurea monotherapy. However, patients with a history of atrial fibrillation, ventricular tachycardia, and ventricular fibrillation were excluded from their study, so their results may not be generalizable to patients with prior arrhythmic conditions. Moreover, younger patients with type 2 diabetes were not captured, as only patients aged >50 years were included in their study, which may lead to biased results. A recent systematic review has shown that type 2 diabetes is increasingly diagnosed in patients aged <50 years in many countries worldwide.^29^ Our study included patients with type 2 diabetes aged ≥40 years, which is more generalizable to a larger population.

Conversely, Eroglu and colleagues^28^ found that sulfonylurea antidiabetics were associated with a lower risk of developing out‐of‐hospital cardiac arrest. However, their study did not match cases and controls by duration of diabetes, which is a risk factor for ventricular arrhythmias.^4^ The duration of diabetes was accounted for during propensity score matching in our study. Moreover, age was not evenly distributed in their study groups, as patients on sulfonylurea drugs alone were older (mean age, 75.2; SD, 9.7) than those on metformin alone (mean age, 69.6; SD, 10.1). Finally, their sample size was small for both patients on sulfonylurea alone (n=215) and patients on metformin alone (n=385), while our study included 16 596 metformin users and 16 596 sulfonylurea users after matching.

### Implications of Subgroup and Sensitivity Analyses

Sensitivity analysis was performed by excluding patients with a history of cardiomyopathy or valvular heart disease, as cardiomyopathies are a common cause of SCD^30^, ^31^; a systematic review identified cardiomyopathies as one of the top causes of SCD in Chinese patients.^32^ Similarly, studies have found associations of aortic valve disease and mitral valve prolapse with VA/SCD.^33^, ^34^, ^35^, ^36^ It was found that the risk for sulfonylurea compared with metformin to develop VA/SCD was similar in both sensitivity and overall analysis.

Common complications of type 2 diabetes include coronary artery disease and myopathy,^37^ which are risk factors for developing VA/SCD.^38^ The risk for these complications increases with the duration and severity of type 2 diabetes, which is consistent with the underlying low‐grade inflammation and glycation.^39^, ^40^ As such, it was possible that the risk of VA/SCD increases with the duration and severity of diabetes, and the influence from medication‐related effects may become less important accordingly. To better elucidate drug‐related effects and minimize confounding by the above factors, we performed a subgroup analysis using insulin usage as a surrogate of diabetic duration and severity. We found that sulfonylurea was consistently associated with greater risk of developing VA/SCD than metformin regardless of diabetes severity, suggesting that the differences between these two drugs are clinically important in patients with diabetes regardless of their condition's severity and duration.

Myocardial ischemia in coronary heart disease alters metabolic and electrical processes in the heart, altering the propagation and conduction of resting and acting membrane potentials, leading to cardiac arrhythmias.^41^ We performed subgroup analyses to explore if coronary heart disease would be a dominant risk factor for VA/SCD such that the differences in arrhythmogenicity between sulfonylurea and metformin would be considered relatively insignificant. The results of the subgroup analyses found the risk of developing VA/SCD was consistently higher in sulfonylurea users compared with metformin users even in patients with coronary heart disease. This further suggested that the differences in arrhythmogenicity between the 2 drugs were significant and clinically important regardless of patients’ inherent risks for VA/SCD.

Subgroup analyses were performed to investigate the risk of VA/SCD among individual types of sulfonylurea. Glicazide, glipizide, tolbutamide, and glimepiride users were at a higher risk of VA/SCD when compared with metformin. Tolbutamide users were at the highest risk of VA/SCD, but this may be attributed to drug‐induced arrhythmias because of a higher usage of proarrhythmic calcium channel blockers and beta blockers in the subgroup.^42^

### Clinical Implications

Although the use of sulfonylurea has decreased in recent years, it remains a commonly prescribed antidiabetic agent, second to metformin.^43^, ^44^ Diabetic nephropathy is a common complication among patients with type 2 diabetes with a prevalence of 31.6% in Hong Kong.^45^ When metformin is contraindicated, such as in patients with severe kidney impairment, sulfonylurea is a viable alternative.^46^, ^47^ The Kidney Disease: Improving Global Outcomes 2020 guidelines recommends patients with an estimated glomerular filtration rate of ≤30 mL/min per 1.73 m^2^ to discontinue metformin therapy^46^, ^48^ However, in practice, the risk of developing VA/SCD by antidiabetic choice is often neglected. Aside from increased mortality risk, VA/SCD necessitates further therapy such as the use of implantable cardioverter‐defibrillators and antiarrhythmic agents and thereby imposes more health care burden.^49^ Patient adherence to metformin may be difficult because of the common side effect of gastrointestinal disturbance,^50^ but this can be avoided with alternative formulations such as extended‐release metformin.^51^ Given the findings of our study, there exists a compelling case to move away from prescribing sulfonylurea for glycemic control.

### Study Limitations

This study has limitations. As this study was retrospective in nature, the effect of unmeasured confounders on the risk of developing VA/SCD cannot be ruled out. For instance, smoking status and alcohol consumption are not recorded by the Clinical Data Analysis and Reporting System. Nonetheless, we have included multiple significant risk factors in the propensity score matching. A proportion of patients were excluded for use of both metformin and sulfonylurea in our study; however, patients on both agents were not included in this comparative study, as they may be suffering from more severe type 2 diabetes compared with patients on either one of the antidiabetics. As patients without hemoglobin A_1c_ values were excluded from the study, the results may not be generalizable to patients with mild type 2 diabetes, who are not necessarily indicated for routine hemoglobin A_1c_ testing. Furthermore, the E‐value suggested that the observed association of the higher VA/SCD risk in sulfonylurea over metformin users would only be insignificant if an unmeasured risk factor exists with an HR of 2.67 to 3.35. A previous study by Chao and colleagues^52^ investigated the risk of VA/SCD in subgroups by comorbidity status, some of which were unmeasured confounders not included in our propensity score model, including end‐stage renal disease (HR, 2.20 [95% CI, 1.91–2.54]), malignancy (HR, 1.98 [95% CI, 1.71–2.31]), autoimmune diseases (HR, 1.93 [95% CI, 1.67–2.22]), and liver cirrhosis (HR, 1.98 [95% CI, 1.61–2.44]). None of these confounders exceeded our lowest E‐value (range, 2.67–3.21). Therefore, the risk of bias caused by unmeasured confounders in our study remains low.

While we have investigated the risk of VA/SCD among different sulfonylureas, the limited use of some sulfonylureas does not allow effective comparisons to be made. Further research comparing risk of VA/SCD between different drugs of the sulfonylurea class is warranted, such that clinicians can avoid choosing select sulfonylureas with high arrhythmogenic risk.

To conclude, this study found that among patients diagnosed with type 2 diabetes, use of sulfonylurea was associated with higher risk of developing VA/SCD compared with use of metformin. The increased risk was consistent in patients with severe diabetes and in those with a history of coronary heart disease. Hence, the use of sulfonylurea should be reconsidered in patients at risk of VA/SCD. Further studies are warranted to study the risk of VA/SCD in different drugs of the sulfonylurea class.

## Sources of Funding

None.

## Disclosures

None.

## Supporting information

Tables S1–S5
Figure S1
