# Supplementary material for: Sulfonylurea Is Associated With Higher Risks of Ventricular Arrhythmia or Sudden Cardiac Death Compared With Metformin: A Population‐Based Cohort Study
Source: J Am Heart Assoc. 2022 Sep 14;11(18):e026289. doi: 10.1161/JAHA.122.026289 (PMC9683657; doi:10.1161/JAHA.122.026289)
Supplement: Supplementary file 1 — Tables S1–S5 Figure S1 [file JAH3-11-e026289-s001.pdf]

SUPPLEMENTAL MATERIAL

Table S1. International Classification of Diseases 9 (ICD-9) codes used for classifying outcomes and comorbidities.

**Medical condition**	**ICD-9 codes**
**Outcome**	
VA/SCD	427.01,427.40-427.59
**Comorbidities**	
Peripheral vascular disease	250.7
Ischemic stroke	433.01,433.11,433.21,433.31, 433.81, 433.91, 434.01, 434.11, 435, 436
Atrial fibrillation	427.31, 429.4
Heart failure	428.00-428.99
Prior VA/SCD	427.01,427.40-427.59
Intracranial hemorrhage	430,431,432.0,432.1,432.9,852.0-852.1,852.2-852.3,852.4-852.5,853.0
Coronary heart disease	410.0-411.9,413.0-414.9
Hypertension	401.9
Chronic Obstructive Pulmonary Disease	491.00-492.99
Cardiomyopathy	425.0-425.9
Valvular disease	424.0-424.4

VA/SCD: Ventricular arrhythmia or sudden cardiac death

Table S2. Baseline characteristics of metformin and sulfonylurea users with missing values for HbA1c.

Characteristics	Metformin (N=31984)	Sulfonylurea (N=22277)
Demographics		
Male, N (%)	13533 (42.3%)	10628 (47.7%)
Baseline age, years	64.84±11.67	69.17±11.87
Follow up duration since type 2 diabetes diagnosis, years	4.77±2.55	4.83±2.56
Comorbidities		
Peripheral vascular disease, N (%)	6 (0.0%)	4 (0.0%)
Ischemic stroke, N (%)	611 (1.9%)	704 (3.2%)
Atrial fibrillation, N (%)	379 (1.2%)	573 (2.6%)
Heart failure, N (%)	462 (1.4%)	804 (3.6%)
Prior VA/SCD, N (%)	1 (0.0%)	6 (0.0%)
Intracranial hemorrhage, N (%)	273 (0.9%)	318 (1.4%)
Coronary heart disease, N (%)	1189 (3.7%)	1295 (5.8%)
Hypertension, N (%)	5190 (16.2%)	4703 (21.1%)
Chronic obstructive pulmonary disease, N (%)	68 (0.2%)	87 (0.4%)
Medications		
ACE inhibitors, N (%)	12114 (37.9%)	8060 (36.2%)
Beta blockers, N (%)	10609 (33.2%)	6913 (31.0%)
Calcium channel blockers, N (%)	13250 (41.4%)	9617 (43.2%)
Diuretics, N (%)	5473 (17.1%)	4185 (18.8%)
Insulin, N (%)	1547 (4.8%)	657 (2.9%)
Thiazolidinediones, N (%)	36 (0.1%)	29 (0.1%)
DPP4 inhibitors, N (%)	7 (0.0%)	4 (0.0%)
GLP-1 agonists, N (%)	0 (0.0%)	0 (0.0%)

Table S3. Baseline characteristics of metformin and sulfonylurea users stratified by insulin versus insulin nonusers after 1:1 propensity score matching.

**Characteristics**	**Insulin users**		**Insulin nonusers**	
	**Metformin (N=2,429)**	**Sulfonylurea (N=2,501)**	**Metformin (N=14,167)**	**Sulfonylurea (N=14,095)**
Demographics				
Male , N (%)	1303 (53.6%)	1359 (54.3%)	6608 (46.6%)	6913 (49.0%)
Baseline age, years	70.73±9.83	71.11±10.86	67.70±11.00	69.33±11.41
Follow up duration since type 2 diabetes diagnosis, years	4.95±2.49	4.93±2.56	4.92±2.55	4.93±2.55
Comorbidities				
Peripheral vascular disease, N (%)	29 (1.2%)	27 (1.1%)	0 (0.0%)	0 (0.0%)
Ischemic stroke, N (%)	156 (6.4%)	204 (8.2%)	703 (5.0%)	782 (5.5%)
Atrial fibrillation, N (%)	138 (5.7%)	194 (7.8%)	596 (4.2%)	772 (5.5%)
Heart failure, N (%)	238 (9.8%)	382 (15.3%)	557 (3.9%)	877 (6.2%)
Prior VA/SCD, N (%)	0 (0.0%)	3 (0.1%)	4 (0.0%)	4 (0.0%)
Intracranial hemorrhage, N (%)	47 (1.9%)	60 (2.4%)	259 (1.8%)	302 (2.1%)
Coronary heart disease, N (%)	441 (18.2%)	537 (21.5%)	1747 (12.3%)	1992 (14.1%)
Hypertension, N (%)	1149 (47.3%)	1359 (54.3%)	4755 (33.6%)	5178 (36.7%)
Chronic obstructive pulmonary disease, N (%)	11 (0.5%)	18 (0.7%)	47 (0.3%)	72 (0.5%)
Medications				
ACE inhibitors, N (%)	1827 (75.2%)	1518 (60.7%)	7021 (49.6%)	7507 (53.3%)
Beta blockers, N (%)	980 (40.3%)	1238 (49.5%)	5858 (41.3%)	5599 (39.7%)
Calcium channel blockers, N (%)	1335 (55.0%)	1450 (58.0%)	6194 (43.7%)	6360 (45.1%)
Diuretics, N (%)	928 (38.2%)	1188 (47.5%)	2833 (20.0%)	3104 (22.0%)
Thiazolidinediones, N (%)	95 (3.9%)	41 (1.6%)	74 (0.5%)	135 (1.0%)
DPP4 inhibitors, N (%)	6 (0.2%)	3 (0.1%)	6 (0.0%)	7 (0.0%)
GLP-1 agonists, N (%)	0 (0.0%)	0 (0.0%)	0 (0.0%)	0 (0.0%)
Laboratory tests				
HbA1c, %	7.44±1.42	7.44±1.45	7.45±1.44	7.45±1.45

Continuous variables were expressed as mean ± standard deviation. VA/SCD: Ventricular arrhythmia or sudden cardiac death; ACE: angiotensin-converting enzyme; DPP4: dipeptidyl-peptidase 4; GLP-1: glucagon-like peptide 1.

Table S4. Baseline characteristics of metformin and sulfonylurea users stratified by history of coronary heart disease (CHD) after 1:1 propensity score matching.

Characteristics	With CHD		Without CHD	
	**Metformin (N=2,188)**	**Sulfonylurea (N=2,529)**	**Metformin (N=14,408)**	**Sulfonylurea (N=14,067)**
Demographics				
Male, N (%)	1144 (52.3%)	1376 (54.4%)	6767 (47.0%)	6896 (49.0%)
Baseline age, years	71.03±9.72	72.46±9.87	67.70±10.99	69.08±11.52
Follow up duration since type 2 diabetes diagnosis, years	5.06±2.51	5.02±2.55	4.90±2.55	4.91±2.55
Comorbidities				
Peripheral vascular disease, N (%)	7 (0.3%)	10 (0.4%)	22 (0.2%)	17 (0.1%)
Ischemic stroke, N (%)	153 (7.0%)	167 (6.6%)	706 (4.9%)	819 (5.8%)
Atrial fibrillation, N (%)	202 (9.2%)	294 (11.6%)	532 (3.7%)	672 (4.8%)
Heart failure, N (%)	309 (14.1%)	583 (23.1%)	486 (3.4%)	676 (4.8%)
Prior VA/SCD, N (%)	4 (0.2%)	5 (0.2%)	0 (0.0%)	2 (0.0%)
Intracranial hemorrhage, N (%)	52 (2.4%)	63 (2.5%)	254 (1.8%)	299 (2.1%)
Hypertension, N (%)	1313 (60.0%)	1558 (61.6%)	4591 (31.9%)	4979 (35.4%)
Chronic obstructive pulmonary disease, N (%)	10 (0.5%)	27 (1.1%)	48 (0.3%)	63 (0.4%)
Medications				
ACE inhibitors, N (%)	1429 (65.3%)	1689 (66.8%)	7419 (51.5%)	7336 (52.2%)
Beta blockers, N (%)	1568 (71.7%)	1686 (66.7%)	5270 (36.6%)	5151 (36.6%)
Calcium channel blockers, N (%)	1071 (48.9%)	1334 (52.7%)	6458 (44.8%)	6476 (46.0%)
Diuretics, N (%)	632 (28.9%)	976 (38.6%)	3129 (21.7%)	3316 (23.6%)
Insulin, N (%)	441 (20.2%)	537 (21.2%)	1988 (13.8%)	1964 (14.0%)
Thiazolidinediones, N (%)	15 (0.7%)	17 (0.7%)	154 (1.1%)	159 (1.1%)
DPP4 inhibitors, N (%)	1 (0.0%)	3 (0.1%)	11 (0.0%)	7 (0.0%)
GLP-1 agonists, N (%)	0 (0.0%)	0 (0.0%)	0 (0.0%)	0 (0.0%)
Laboratory tests				
HbA1c, %	7.50±1.42	7.51±1.50	7.44±1.44	7.44±1.45

Continuous variables were expressed as mean ± standard deviation. VA/SCD: Ventricular arrhythmia or sudden cardiac death; ACE: angiotensin-converting enzyme; DPP4: dipeptidyl-peptidase 4; GLP-1: glucagon-like peptide 1.

Table S5. Baseline characteristics of individual sulfonylurea users after 1:1 propensity score matching.

**Characteristics**	**Glicazide (N=10116)**	**Glipizide (N=399)**	**Tolbutamide (N=302)**	**Glibenclamide (N=1216)**	**Glimepiride (N=180)**
Demographics					
Male, N (%)	4942 (48.9%)	200 (50.1%)	144 (47.7%)	633 (52.1%)	97 (53.9%)
Baseline age, years	70.05±11.40	72.66±11.77	73.34±11.29	67.36±10.30	69.42±11.05
Follow up duration since type 2 diabetes diagnosis, years	4.94±2.54	5.01±2.59	4.98±2.56	5.06±2.67	4.61±2.57
Comorbidities					
Peripheral vascular disease, N (%)	0 (0.0%)	0 (0.0%)	0 (0.0%)	0 (0.0%)	0 (0.0%)
Ischemic stroke, N (%)	648 (6.4%)	27 (6.8%)	20 (6.6%)	32 (2.6%)	9 (5.0%)
Atrial fibrillation, N (%)	622 (6.1%)	22 (5.5%)	32 (10.6%)	36 (3.0%)	3 (1.7%)
Heart failure, N (%)	729 (7.2%)	33 (8.3%)	34 (11.3%)	28 (2.3%)	10 (5.6%)
Prior VA/SCD, N (%)	4 (0.0%)	0 (0.0%)	0 (0.0%)	0 (0.0%)	0 (0.0%)
Intracranial hemorrhage, N (%)	244 (2.4%)	13 (3.3%)	6 (2.0%)	19 (1.6%)	4 (2.2%)
Coronary heart disease, N (%)	1492 (14.7%)	71 (17.8%)	60 (19.9%)	143 (11.8%)	21 (11.7%)
Hypertension, N (%)	3968 (39.2%)	170 (42.6%)	187 (61.9%)	355 (29.2%)	60 (33.3%)
Chronic obstructive pulmonary disease, N (%)	69 (0.7%)	0 (0.0%)	1 (0.3%)	1 (0.1%)	0 (0.0%)
Medications					
ACE inhibitors, N (%)	5457 (53.9%)	227 (56.9%)	167 (55.3%)	587 (48.3%)	116 (64.4%)
Beta blockers, N (%)	4025 (39.8%)	173 (43.4%)	163 (54.0%)	427 (35.1%)	91 (50.6%)
Calcium channel blockers, N (%)	4672 (46.2%)	203 (50.9%)	214 (70.9%)	494 (40.6%)	102 (56.7%)
Diuretics, N (%)	2362 (23.3%)	119 (29.8%)	126 (41.7%)	182 (15.0%)	64 (35.6%)
Insulin, N (%)	495 (4.9%)	37 (9.3%)	36 (11.9%)	31 (2.5%)	44 (24.4%)
Thiazolidinediones, N (%)	81 (0.8%)	8 (2.0%)	1 (0.3%)	7 (0.6%)	12 (6.7%)
DPP4 inhibitors, N (%)	3 (0.0%)	0 (0.0%)	0 (0.0%)	0 (0.0%)	0 (0.0%)
GLP-1 agonists, N (%)	0 (0.0%)	0 (0.0%)	0 (0.0%)	0 (0.0%)	0 (0.0%)

Laboratory tests					
HbA1c, %	7.45±1.46	7.54±1.56	7.62±1.52	7.40±1.44	7.53±1.47

VA/SCD: Ventricular arrhythmia or sudden cardiac death; ACE: angiotensin-converting enzyme; DPP4: dipeptidyl-peptidase 4; GLP-1: glucagon-like peptide

Figure S1. Covariate balance before and after propensity score matching.

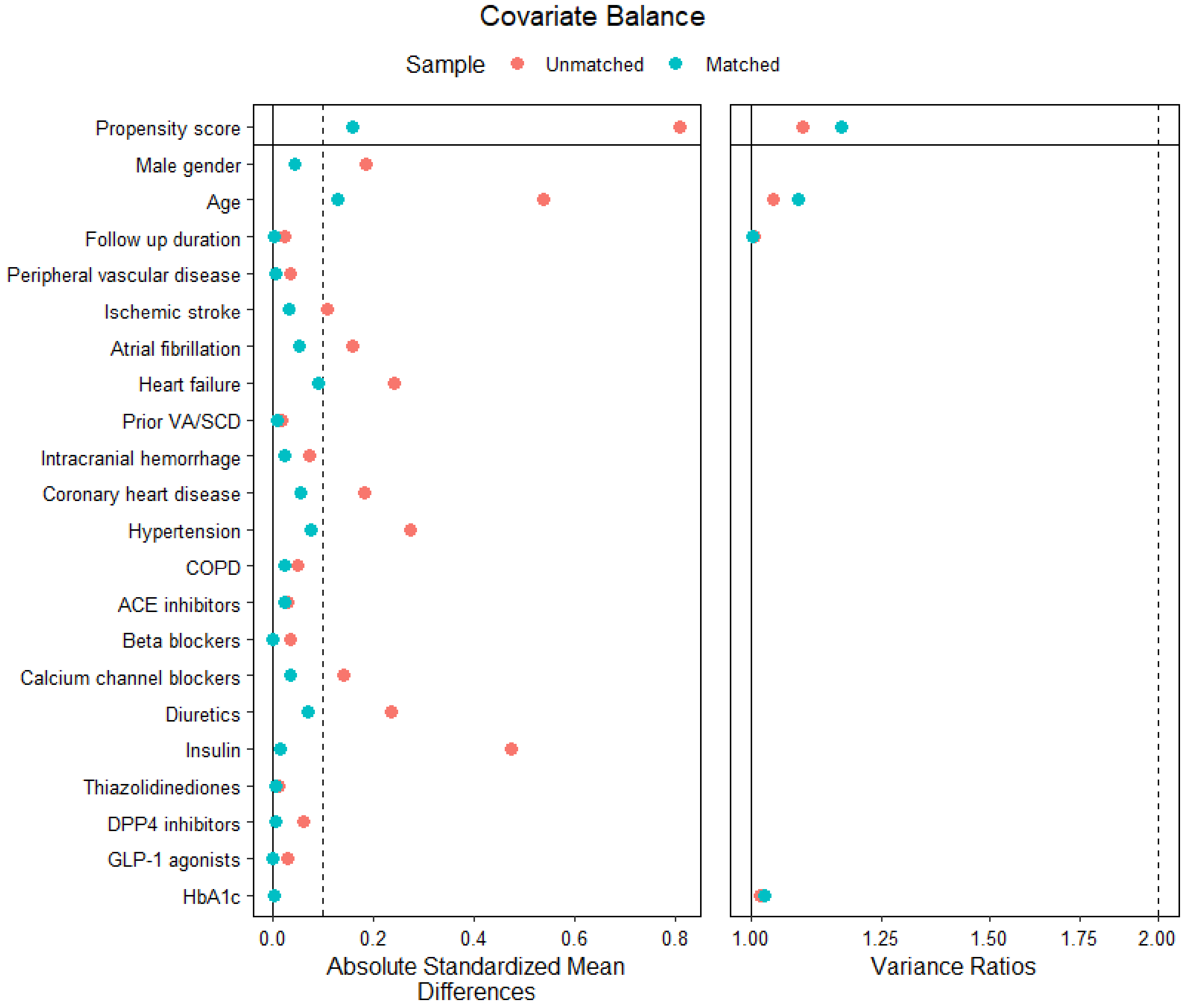

VA/SCD: Ventricular arrhythmia or sudden cardiac death; ACE: angiotensin-converting enzyme; COPD: chronic obstructive pulmonary disease; DPP4: dipeptidyl-peptidase 4; GLP-1: glucagon-like peptide 1.
